# Bistability, Probability Transition Rate and First-Passage Time in an Autoactivating Positive-Feedback Loop

**DOI:** 10.1371/journal.pone.0017104

**Published:** 2011-03-21

**Authors:** Xiu-Deng Zheng, Xiao-Qian Yang, Yi Tao

**Affiliations:** 1 Key Laboratory of Animal Ecology and Conservational Biology, Centre for Computational and Evolutionary Biology, Institute of Zoology, Chinese Academy of Sciences, Beijing, People's Republic of China; 2 School of Mathematical Sciences, Beijing Normal University, Beijing, People's Republic of China; 3 Graduate University of the Chinese Academy of Sciences, Beijing, People's Republic of China; University of Milano-Bicocca, Italy

## Abstract

A hallmark of positive-feedback regulation is bistability, which gives rise to distinct cellular states with high and low expression levels, and that stochasticity in gene expression can cause random transitions between two states, yielding bimodal population distribution (Kaern et al., 2005, *Nat Rev Genet 6: 451-464*). In this paper, the probability transition rate and first-passage time in an autoactivating positive-feedback loop with bistability are investigated, where the gene expression is assumed to be disturbed by both additive and multiplicative external noises, the bimodality in the stochastic gene expression is due to the bistability, and the bistability determines that the potential of the Fokker-Planck equation has two potential wells. Our main goal is to illustrate how the probability transition rate and first-passage time are affected by the maximum transcriptional rate, the intensities of additive and multiplicative noises, and the correlation of additive and multiplicative noises. Our main results show that (i) the increase of the maximum transcription rate will be useful for maintaining a high gene expression level; (ii) the probability transition rate from one potential well to the other one will increase with the increase of the intensity of additive noise; (iii) the increase of multiplicative noise strength will increase the amount of probability in the left potential well; and (iv) positive (or negative) cross-correlation between additive and multiplicative noises will increase the amount of probability in the left (or right) potential well.

## Introduction

Bistability arises within a wide range of biological systems from the bacteriophage 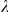 to cellular signal transduction pathways in mammalian cells [1], [2]. As a fundamental behavior of biological system, bistability has been studied extensively through experiments, theoretical analysis and numerical simulations. Hasty et al. [3] considered a single network derived from bacteriophage 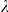 and constructed a two-parameter deterministic model describing the temporal evolution of the concentration of 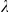 repressor protein. They showed how additive and multiplicative external noise can be used to regulate gene expression. In the case with only additive noise, they demonstrated the utility of such control through the concentration of protein switch, whereby protein production is turned “on” and “off” by using short noise pulse. In the case with multiplicative noise, they showed that small deviations in the transcription rate can lead to large fluctuations in the production of protein. Combining theory and experiments, Isaacs et al. [4] investigated the dynamics of an isolated genetic module, an *in vivo* autoregulatory gene network. As predicted by their theoretical model, temperature-induced protein destabilization led to the existence of two expression states. The result of Isaacs et al. shows clearly the effects of varying the strength of feedback activation on population heterogeneity (see also [5]). Recently, Acar et al. [6] experimentally explored how switching affects population growth by using the galactose utilization network of *Saccharomyces cerevisiae*.

Kaern et al. [2] pointed out that a hallmark of positive-feedback regulation is bistability, which gives rise to distinct cellular states with high and low expression levels, and that stochasticity in gene expression can cause random transitions between the two states, yielding bimodal population distributions (see also [7]–[9]). So, for the positive-feedback regulation with bistability, a challenging question is how to determine probability transition rates between the two states, or how the random transitions between the two states are affected by the transcription rate and noise strength. In this paper, a simple theoretical model for an autoactivating positive-feedback loop is investigated. Our main goal is to provide a theoretical analysis for the probability transition rate and first-passage time in the system with external noise. The paper is organized as follows. In section 2, the basic model and its bistability is presented. The analysis of the probability transition rate and first-passage time for the situations with additive and multiplicative external noises are given in section 3.

## Results

### Basic model

**Figure 1:**
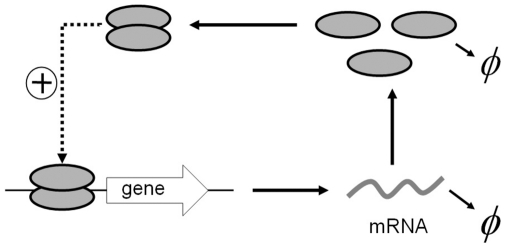
Modeling of autoactivating positive-feedback loop. In this model, the transcriptional activator monomers bind into dimers that bind to specific DNA sequences near the promoter, activating production of the activator monomers. The dotted line represents the positive auto-regulation. The degradation of both mRNA and protein is denoted by the slashed circle.

It is well known that the simplest circuit motif able to exhibit multiple stable states is the autoactivating positive-feedback loop [5], [9]–[11], in which a single gene encodes a protein (activator), and the activator monomers bind into dimers that subsequently bind to the upstream regulatory site of the gene, activating production of the activator monomers (see Figure 1). For example, the autoactivation of CI protein by the 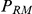 promoter of phage 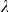 [4]. The autoactivating positive-feedback loop is expected to exhibit bistability for the protein synthesis level, i.e., a higher level and a low level of protein concentration [2], [9]. Let 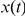 and 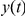 denote the concentrations of mRNA and activator protein at time 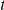, respectively. Then, in general the macroscopic rate equation for 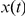 and 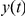 can be expressed as
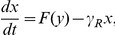


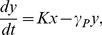
(1)where 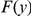 represents the mRNA transcription rate, which is defined as a function of activator protein concentration with 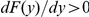, the parameter 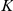 denotes the translation rate, and the parameters 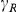 and 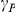 are the degradation rates of mRNA and protein, respectively, with 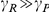, i.e., the concentration of mRNA is a fast variable compared with the concentration of activator protein [12], [13]. For the mRNA transcription rate 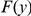, we take it as a Hill-type function
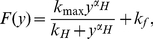
(2)where 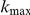 is the maximum transcription rate, 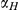 the Hill coefficient where we take 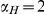, 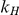 the Hill constant, and 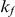 the basal transcription rate with 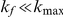 [14]. In biology, these parameters mean that: (i) 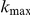 represents the mRNA transcription rate when the activator protein concentration is large enough; (ii) 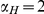 implies that the activator binding processes are considered comparatively rapid and close to equilibrium, so the concentration of activator homodimer is proportional to the square of activator monomer concentration [14]; (iii) 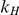 is the dissociation constant of activator dimer from the regulatory site; and (iv) 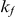 is the mRNA transcription rate when the activator protein concentration is very low [15].

Notice that 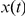 can be considered to be a fast variable compared with 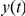 since 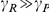. Then, Eq. 1 can be reduced as
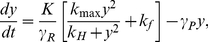
(3)i.e., the fast variable can be assumed to be at an effective equilibrium, whereas the slow variable is responsible for the dynamics of the system [5], [16], [17]. In mathematics, one of the most important properties of Eq. 3 is its bistability, i.e., Eq. 3 has at most three fixed points, denoted by 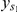, 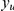 and 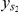 with 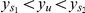. For the stability of 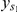, 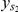 and 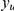, it is easy to see that both 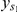 and 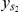 are locally asymptotically stable and 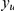 is unstable since 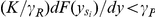 for 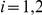 and 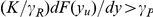 (see also [15]).

**Figure 2:**
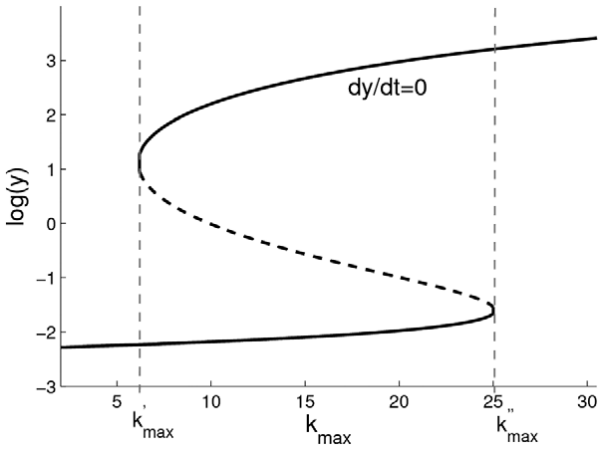
Bifurcation analysis of bistability in the deterministic gene expression as a function of 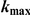. The parameters 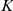, 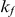, 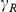, 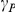 and 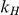 are taken as 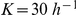, 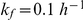, 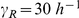, 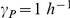 and 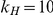 (see also the main text). The bistability exists if 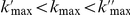, where 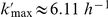 and 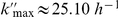). If 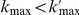 (or 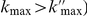, then the system will be monostable (see also Ref. [14]).

In biology, we are more interested in how the dynamic properties of Eq. 3 is affected by the maximum transcription rate 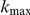 (see also [15]). The relationship between the bistability and 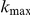 is plotted in Figure 2 (i.e. 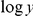 vs. 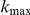 for 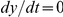) where, following Smolen et al. [15], the parameters are taken as 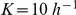, 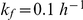, 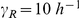, 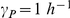 and 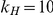 (see also [16]) (in this paper, we keep these parameters to be fixed). Clearly, for the stability of Eq. 3, the parameter 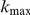 has two bifurcation values, denoted by 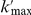 and 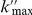, respectively, with 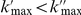 (where 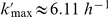 and 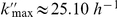), i.e., the bistability exists if 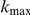 is in the interval 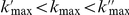. For the situation with 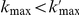 (or 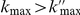), the system will be monostable, i.e., the system has only one fixed point 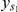 (or 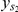) if 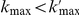 (or if 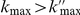). On the other hand, if 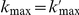 (or 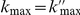), then the system will have two fixed points 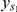 and 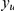 (or 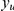 and 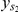), i.e., if 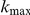 exactly equal its bifurcation value, then Eq. 3 will have two fixed points, in which one is stable and the other semi-stable.

Similar to Hasty et al. [3], we here consider also how the dynamics of protein concentration is affected by the additive and multiplicative external noises. As discussed above, if the bistability exists, then in the absence of noise, the system state will evolve identically to one of the two fixed points. The presence of a noise source will lead to the fluctuation of the system state. Hasty et al. [3] pointed out that an additive noise source alters the “background” protein production. This means that we need to consider the effect of a randomly varying external field on the biochemical reactions. For example, To et al. [18] provided an experiment evident to show that the change of the external noise can induce bimodality in positive transcriptional feedback loops without bistability. On the other hand, Hasty et al. [3] also pointed out that although transcription is represented by a single biochemical reaction, it is actually a complex sequence of reactions, and it is natural to assume that this part of the gene regulatory sequence is likely to be affected by fluctuations of many internal or external parameters. This implies that the transcription rate can be also considered to be a random variable.

In our model, the additive noise, denoted by 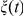, alters the “background” protein production, and is defined as a white noise with 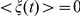 and 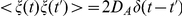 where 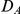 measures the level of additive noise strength. The multiplicative noise alters the transcription rate. We vary the transcription rate by allowing the parameter 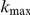 to vary stochastically, i.e., let 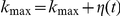, where 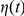 is also a white noise with 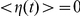 and 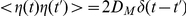 where 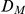 measures the level of multiplicative noise strength. Here a natural question is whether the additive and multiplicative noises are statistically correlated. However we could imagine the correlation arising from the feedback regulation, i.e. the transcription rate (affected by noise) is chemically coupled to the protein concentration (also affected by noise). We define that the cross-correlation of 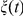 and 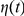 is 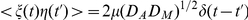 where 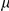 is the cross-correlation intensity [19]. In fact, for the cross-correlation between the additive noise 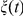 and multiplicative noise 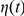, we have no experimental evidence that indicate that the parameter 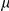 should be positive, or negative. We also noticed that in a previous model developed by Hasty et al. [3], the effect of the cross-correlation between the additive and multiplicative noises on the stochastic gene expression is ignored. Thus, for the effect of 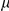 we will only provide some theoretical possibilities.

According to the above definitions about 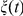 and 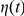, the Langevin equation corresponding to Eq. 3 is given by
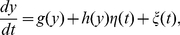
(4)where
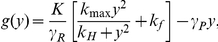


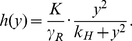
(5)


Let 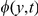 denote the probability density distribution that the concentration of activator protein exactly equals 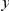 at time 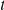. Then, from Risken [20], the Fokker-Planck equation of 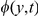 corresponding to Eq. 4 can be given by
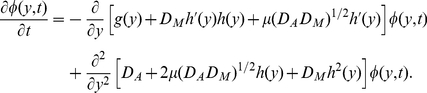
(6)The stationary distribution is 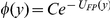 where 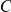 is the normalized constant and
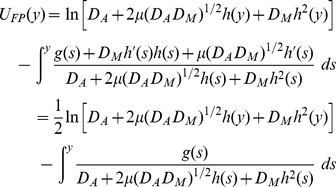
(7)is called the potential of the Fokker-Planck equation.

### Additive noise

In this subsection, we consider only the effect of additive noise on the bistability, and assume that 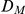 = 0 (i.e. we here ignore the multiplicative noise). Clearly, for 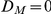, Eq. 6 can be reduced to
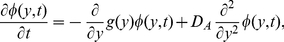
(8)and Eq. 7 can be rewritten as
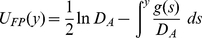
(9)with 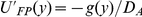. The stationary solution of Eq. 8 is 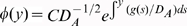 where 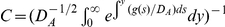. Obviously, 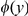 is a bimodal distribution if 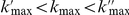, i.e., stationary distribution 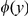 has two peaks corresponding to the two stable points 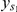 and 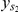, respectively. This also implies that the stable point 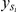 (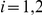) must correspond to the local minimum of the potential 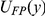, and the unstable point 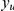 to the local maximum of 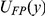. In physics, the local minimum of 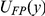 corresponding to 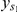 (or 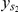) is also called the potential well, and the local maximum of 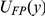 corresponding to 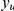 the potential barrier [20]. Thus, for convenience we call the potential well corresponding to 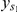 (or 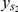) the left (or right) well.

**Figure 3:**
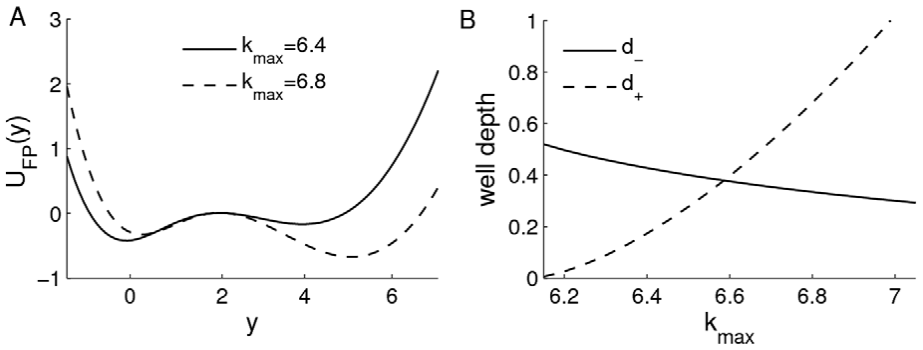
Effect of 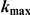 on the potential 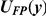. **A**) The potential for different 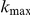 values, where 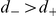 (solid curve) with 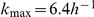 and 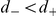 (dotted curve) with 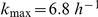. **B**) The relationship between the depth of potential well and 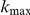. When 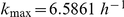, both right and left potential wells have the same depth, i.e., 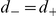.

The depth of the right well is defined as 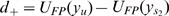, and, similarly, the depth of the left well is 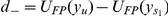. The depth of the potential well varies as the function of 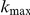 where we take the parameters 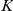, 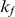, 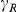, 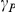 and 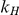 to be fixed. The bistable potential 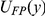 is plotted in Figure 3A for two different 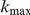 values, where 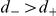 (solid curve) with 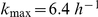 and 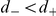 (dotted curve) with 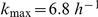. This strongly implies that the depth of left well decreases with the increase of 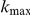 but the depth of right well increases with the increase of 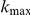. The relationship between the depth of potential well and 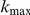 is plotted in Figure 3B, i.e. the system state should be more easily attracted by the left well with the increase of the maximum transcription rate. It is also easy to see that there must exist a 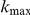 value such that 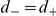 (in Figure 3B, 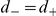 if 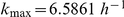).

**Figure 4:**
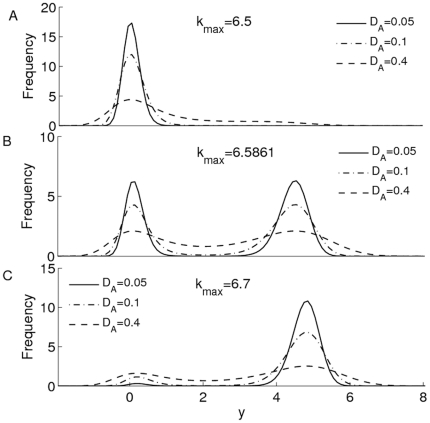
Effect of 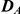 on 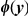. The effects of additive noise strength 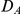 on the stationary distribution 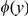 for different 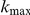 values are plotted, where 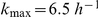 in **A**, 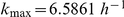 in **B** and 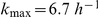 in **C**.

The effects of 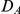 and 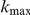 on the stationary distribution are plotted in Figure 4. We show that the decrease of 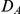 will increase the total probability in the left well if 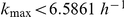 (with 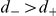) and the total probability in the right well if 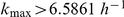 (with 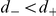). The stationary distribution 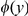 with 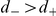 (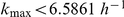), 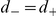 (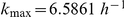) and 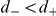 (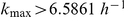) are plotted in Figure 4A, 4B and 4C, respectively. We noticed that this result has been used to explain how the external noise can be used to control the level of protein synthesis [2], [3]. For example, Hasty et al. investigated the autoregulation of bacteriophage 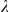 repressor expression network which contains three operator sites known as OR1, OR2, and OR3 and constructed a two-parameter deterministic model describing the temporal evolution of the concentration of 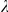 repressor protein [3]. They showed how the bistable regime is enhanced with the addition of the first operator site in the promoter region through comparing two models with only the last two operator sites and the full operator regions, respectively. They also showed how external noise can be used to regulate expression through adding external additive noise or multiplicative noise using the stochastic simulations. For the case with additive noise, they demonstrated the utility of such control through the successful switch of the concentration of a protein, whereby protein production is turned “on” and “off” by using short noise pulses, where the short noise pulse means that the noise of the system is rapidly increased to a high level in a short period of time, and after this pulse, the noise is returned to its original value.

When the system state is near the stable point 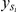 (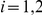), the steady-state statistics of the system can be given by 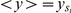 and 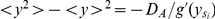 (the proof is given in Text S1) (see also [21]). This result shows clearly that when the system state is near the stable point 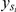, the intensity of stochastic fluctuations around 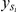 is proportional to the noise strength 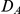 but inversely proportional to 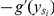. On the other hand, for the bistable potential function 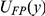, a more challenging question is how the system state jumps from one potential well to the other (i.e. the state is switches from one well to the other) because of the external noise.

**Figure 5:**
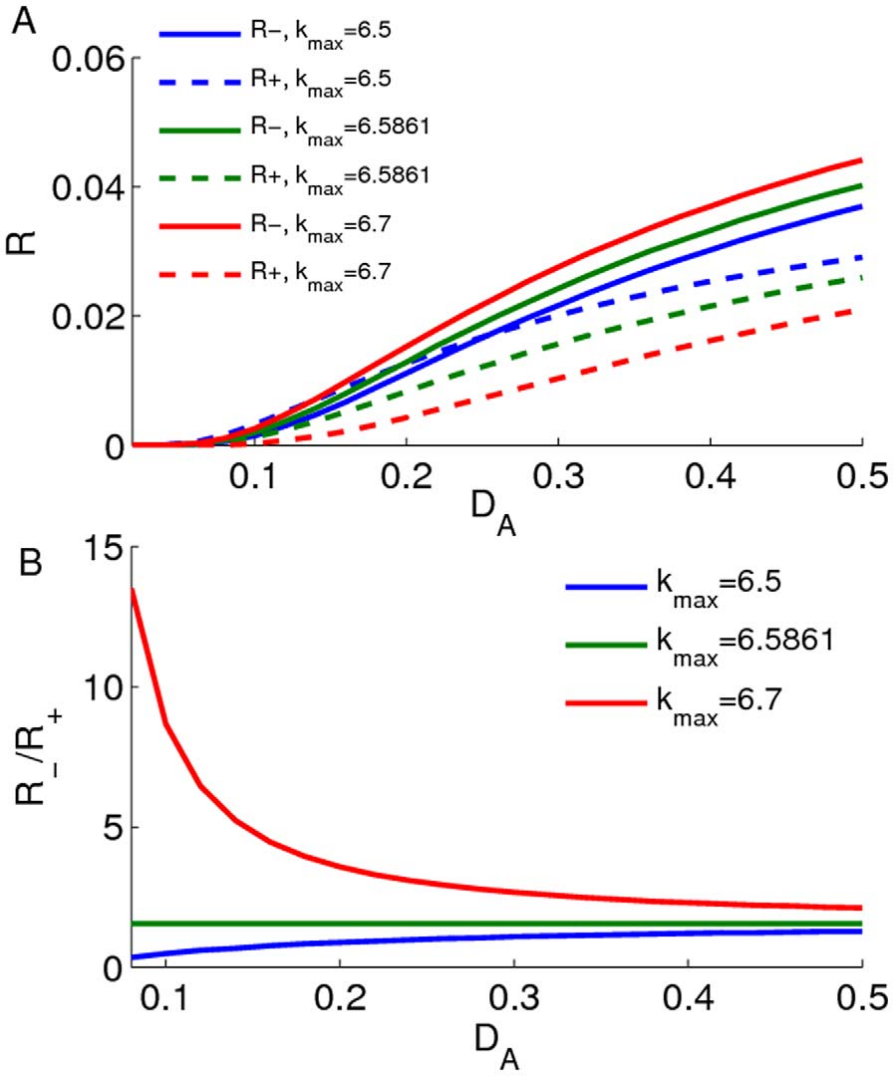
Effect of 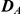 on probability transition rate. The effects of additive noise strength 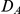 on both probability transition rates 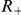 and 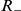 are shown: **A**) For different 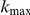 values, both 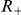 and 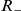 will increase with the increase of 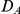. **B**) The ratio of 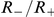 will increase (or decrease) with the increase of 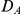 if 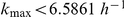 (or 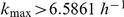). For 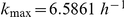, the ratio 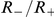 is a constant.



Notice that the probability exchange between two potential wells occurs only near the potential barrier (i.e., at the unstable point 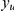). Thus, the increase (or decrease) of the amount of probability in one potential well must result in the decrease (or increase) of the amount of probability in the other. Let 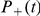 denote the total probability in the right well at time 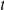, i.e., 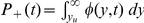, and, similarly, 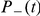 the total probability in the left well at time 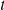, i.e., 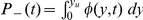 (where we must have 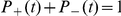). Then the master equations of 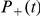 and 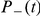 can be given by
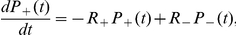


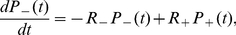
(10)where 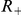 is the probability transition rate from the right well to the left well, and 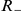 the probability transition rate from the left well to the right well, which are given by
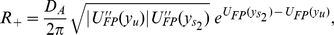


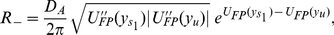
(11)respectively, (the mathematical derivation is given in Text S1) (see also [22]). Obviously, Eq. 10 has two eigenvalues, one is 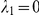, and the other 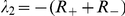. The first one is the eigenvalue of the Fokker-Plack equation corresponding to the stationary distribution, and the latter is the lowest non-vanishing eigenvalue where 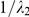 measures the largest time scale of the probability transition between two potential wells. From Eq. 11, we have that:

1. For both 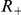 and 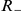, we have 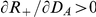 and 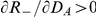. This means that the increase of 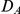 will promote the probability exchange between two wells (see Figure 5A).
2. The transition rate will decrease with the increase of the well depth, i.e., 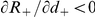 and 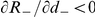 (where 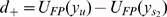 and 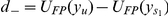). Hence, from Figure 3B, we have also that 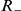 will increase with the increase of 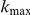 but 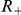 will decrease with the increase of 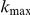. In biology, this means that the increase of 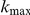 will promote the probability transfer from the left well to the right well, or the increase of 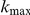 will be useful for maintaining a high gene expression level (see also Figure 4).
3. For convenience, we use ratio 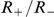

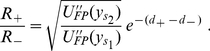
(12)to measure the relative intensity of probability exchange between two wells, i.e., if 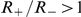, then the probability is more easily transferred from the right well to the left well, and, conversely, if 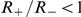, then the probability is more easily transferred from the left well to the right well. The effect of noise strength on the ratio 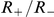 mainly depends on the difference between the depths of right and left wells (i.e., 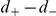) since we have that 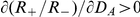 if 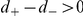, and 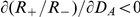 if 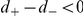. This result shows clearly that the increase of 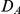 will promote probability exchange from the deep well to the shallow well. The relationship between 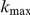, 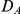 and 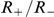 is plotted in Figure 5B. Clearly, the ratio 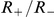 is an increasing function of 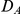 if 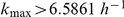 (where 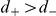), and a decreasing function of 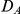 if 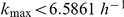 (where 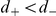). Particularly, when 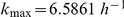, the ratio 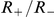 keeps a constant (where 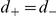), i.e., it is independent of 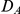. On the other hand, it is also easy to see that the equilibrium solution of Eq. 10 must satisfy 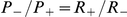, i.e. 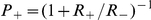 and 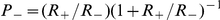. This shows clearly how the probabilities in the right and left wells are affected by 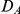 through the ratio 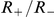.

In physics, the first-passage time is defined as the time at which the stochastic variable first leaves a given domain [20]. In general, the first-passage time can be used to measure the robustness of the system steady-state. For our model, let 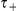 (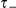) denote the first-passage time at which the system state first leaves the right (left) well across the potential barrier. Under the weak noise (i.e., 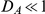), the expectations of 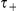 and 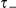 can be approximated as
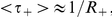


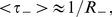
(13)respectively, and the variances of 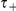 and 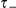, denoted by 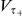 and 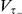, are
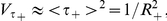


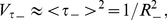
(14)respectively (the mathematical derivations of Eqs 13 and 14 are given in Text S1). This strongly implies that under the weak noise, both 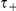 and 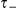 should approximately obey the exponential distribution. Statistically, all transition events (called also the escape events) in a given direction (for example, from the left well to the right well, or from the right well to the left well) can be roughly considered to be independent of each other with a given average rate, i.e. the number of the transition events in a given time interval should be a Poisson process. Thus, the distribution of the first-passage time should be an exponential distribution, and its scale parameter is the inverse of the probability transition rate [23].

The numerical simulation results for the statistical properties of 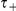 and 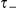 are given in Table 1 (The simulation algorithm is given in Text S1). It is easy to see that for different 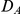 values (where we take 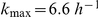), the average of 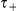 (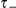) (i.e. the mean first-passage time) and its standard deviation are almost same. The Monte Carlo simulations also show that under the weak noise, the relation 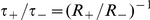 is true (see Table 2). All of these simulation results exactly match the theoretical predictions.

**Table 1:** The Monte Carlo simulation results for the effect of additive noise strength 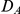 on the statistical properties of first passage time 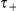 and 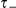 with 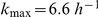 (FPT: first-passage time).

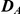	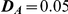		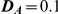		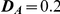		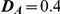	
FPT	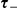	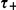	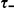	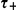	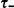	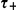	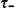	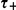
MEAN( 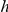 )	11319	28116	285.00	569.79	42.16	78.57	13.66	25.06
SD( 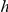 )	11123	27295	280.95	552.35	40.26	78.47	12.91	23.76

**Table 2:** The Monte Carlo simulation results for both ratios 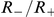 and 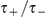 with different values of 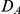 and 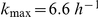.

Ratio	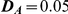	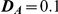	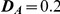	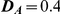
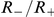	2.45	2.00	1.80	1.70
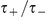	2.48	2.00	1.86	1.83

### Multiplicative noise

**Figure 6:**
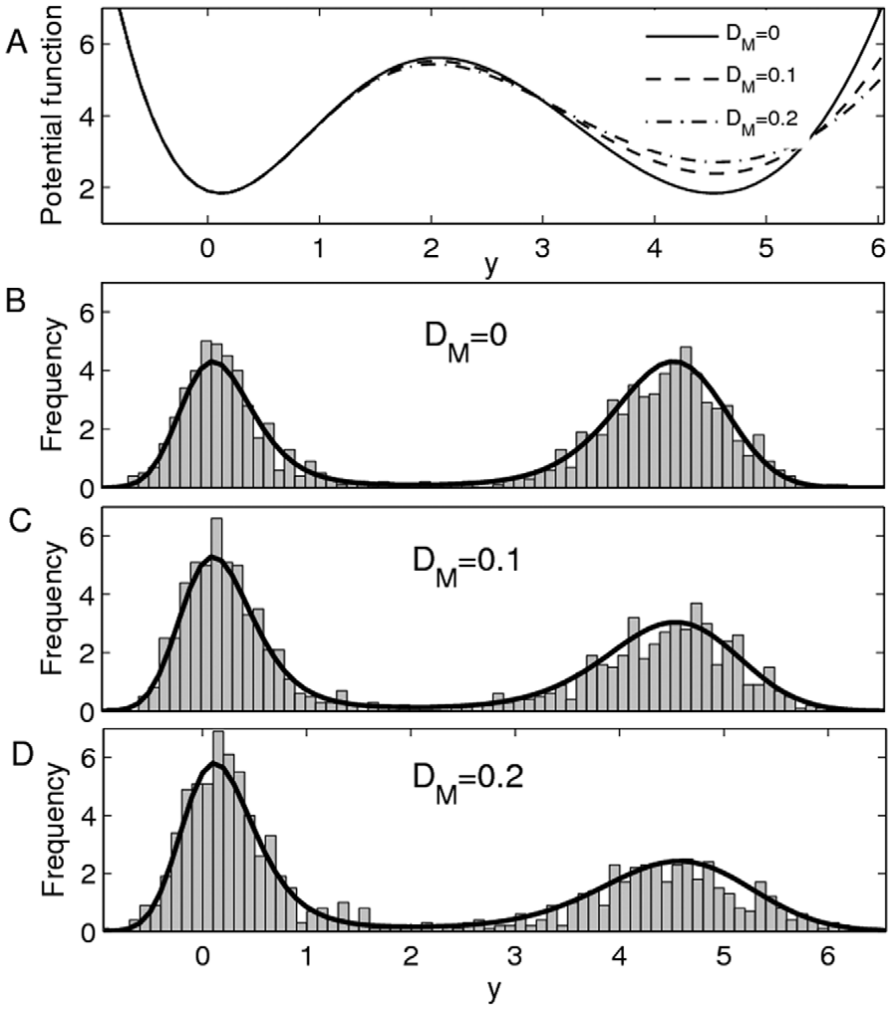
Effect of 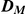 on 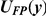 and 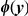. The effects of multiplicative noise strength 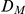 on the potential 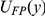 and stationary distribution 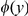 with 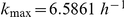 and 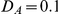 are shown: **A**) The depth of the right potential well will decrease with the increase of 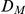, but the depth of the left potential well is not sensitive to the change of 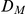. **B–D**) The Monte Carlo simulation results for different 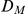 values, i.e., 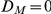 in **B**, 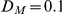 in **C** and 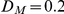 in **D**, where the solid curves denote the theoretical stationary distribution.

For 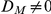, we first consider the situation with 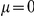, i.e., 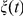 and 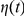 are independent of each other. According to this definition, we have that
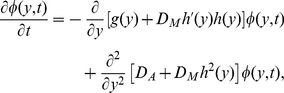
(15)and that
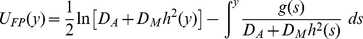
(16)(see Eqs. 6 and 7). In this situation, the effect of 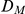 on 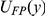 is plotted in Figure 6A, where the parameters 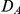 and 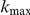 are taken as 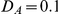 and 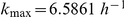. In subsection 3.1, we have shown that for 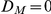, both right and left wells have the same depth if 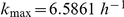. We will select the special value in the following analysis. We can find that the depth of the right well will decrease with the increase of 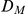, but the change in the depth of the left well is very small. The stationary distribution and the Monte Carlo simulation corresponding to Figure 6A are plotted in Figure 6B–6D, respectively. These results show clearly that the amount of probability in the left (right) well will increase (decrease) with the increase of 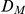.

Similar to the analysis in subsection 3.1, if both additive and multiplicative noises are weak, i.e., 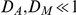, the the expectations of the first-passage times 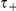 and 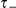 can be approximated as
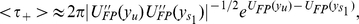


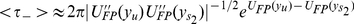
(17)(the mathematical derivation is given in Text S1) (see also [20], [22]). The Monte Carlo simulation results for the statistical properties of 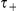 and 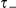 are given in Table 3, in which we can find that not only both 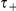 and 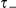 should obey the exponential distribution but also 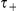 is more sensitive for 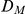 than 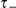. This implies that the increase of the multiplicative noise strength will be useful for maintaining the protein concentration at the low level.

**Table 3:** The Monte Carlo simulation results for the effect of multiplicative noise strength 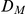 on the statistical properties of first passage time 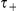 and 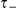 with 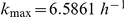 and 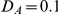.

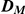	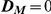		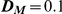		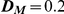		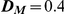	
FPT	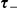	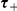	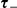	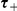	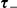	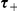	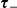	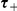
MEAN( 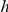 )	279.15	463.91	270.29	196.81	264.05	114.63	246.62	56.30
SD( 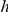 )	285.05	469.25	267.15	189.51	262.32	108.61	239.91	52.95

**Figure 7:**
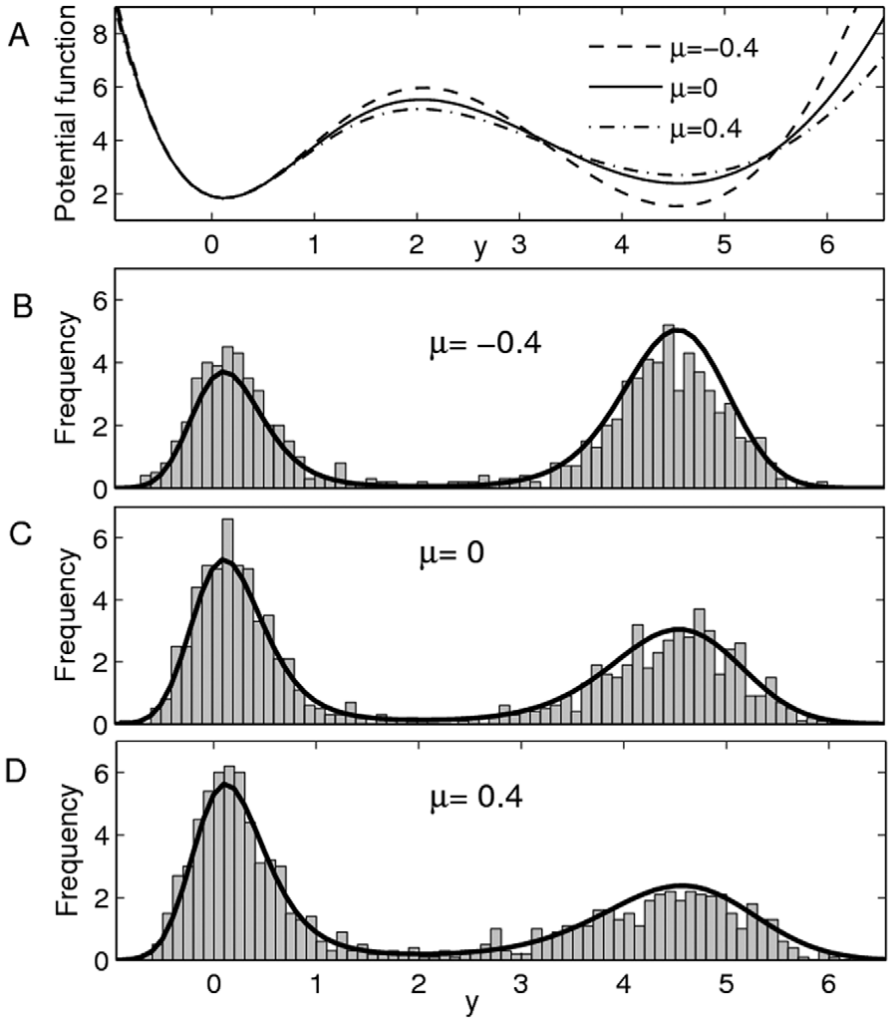
Effect of 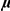 on 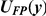 and 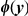. The effects of 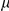 on the potential 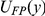 and stationary distribution 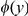 with 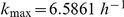, 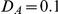 and 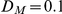 are shown: **A**) The negative (or positive) correlation between additive and multiplicative noises will increase (or decrease) the depth of the right potential well. The effect of 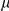 on the left potential well is very small. **B–D**) The Monte Carlo simulation results for different 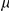 values, i.e., 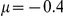 in **B**, 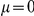 in **C** and 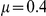 in **D**, where the solid curves denote the theoretical stationary distribution. Clearly, the negative (or positive) correlation will increase the probability in the right (or left) potential well.

Secondly, for 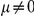, we are interested in how the stochastic dynamics of the system is affected by the correlation between 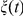 and 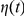. The effect of 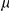 on 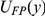 is plotted in Figure 7A where 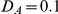, 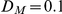 and 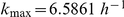, in which we can also find that the depth of the right well is more sensitive for the change of 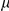 than the depth of the left well, and that if 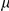 is negative, then the depth of the right well will increase with the increase of 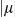 (i.e. absolute value of 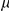), and, conversely, if 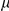 is positive, the depth of the right well will decrease with the increase of 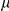. The effect of 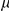 on the stationary distribution and the Monte Carlo simulation corresponding to Figure 7A are plotted in Figure 7B–7D. It shows clearly that the positive correlation (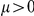) will increase the amount of probability in the left well, and, conversely, the negative correlation (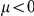) will increase the amount of probability in the right well.

**Figure 8:**
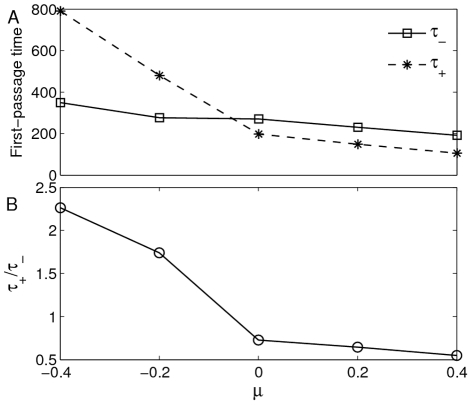
Effect of 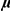 on first-passage time. The Monte Carlo simulation for the effect of 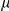 on the first passage time 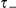, 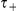 and ratio 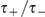 with 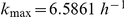, 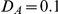 and 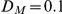. **A**) Both 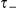 and 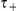 will decrease with the increase of 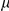. **B**) The ratio 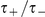 also decreases with the increase of 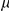.

For the effect of 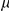 on the first-passage time, the Monte Carlo simulation results are showed in Figure 8, in which both 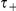 and 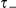 will decrease with the increase of 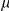, and the change rate of 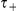 is obviously larger than that of 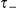. We can also notice that the ratio 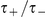 will decrease with the increase of 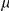 (see Figure 8B). This also implies that the positive (or negative) correlation between 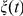 and 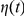 will promote the probability transition from the right (left) well to the left (right) well (see also Figure 7B–7D). Clearly, the dependence of the ratio 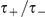 on 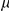 reflects how the stationary distribution is influenced by the cross-correlation between additive and multiplicative noises, or, theoretically, the cross-correlation between 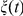 and 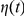 should be also used to control the protein synthesis.

## Discussion

In this paper, the probability transition rate and first-passage time in an autoactivating positive-feedback loop with bistability are investigated. In our model, similar to Hasty et al. [3], the gene expression is assumed to be disturbed by both additive and multiplicative external noises, and the bimodality in the stochastic gene expression is due to the bistatility. The bistability of the deterministic dynamics Eq. 3 implies that the potential of the Fokker-Planck equation Eq. 6 has two potential wells, which correspond to the two stable points of Eq. 3, respectively, and that the stationary solution (i.e. stationary distribution) of the Fokker-Planck equation is a bimodal distribution. For our main goal, we are interested in how the system state jumps from one potential well to the other because of the external noise. In subsection 3.1, for the situation with only additive noise, our main results show that (i) both probability transition rates 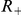 and 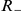 will increase with the increase of 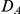; (ii) 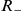 will increase with the increase of 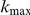 but 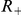 will decrease with the increase of 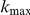, i.e., the increase of 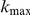 will be useful for maintaining a high gene expression level; (iii) the ratio 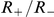 measures the relative intensity of probability exchange between two potential wells, and there is a critical value of 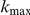 (which is 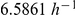 in our case) such that the ratio 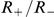 is an increasing function (or a decreasing function) of 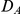 if 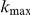 is larger (or smaller) than the critical value; and (iv) for both first-passage times 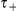 and 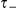, if 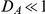 (i.e. the additive noise is weak), then they obey the exponential distribution with expectations 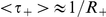 and 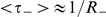, respectively. In subsection 3.2, for the situation with both additive and multiplicative noises, we show that (i) for 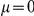 (i.e. the additive noise and multiplicative noise are independent of each other), the increase of 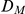 will be useful for maintaining the protein concentration at the low level; and (ii) for 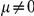, the positive (or negative) correlation between additive and multiplicative noises will promote the probability transition from the right (left) well to the left (right) well, i.e., the positive correlation will increase the amount of probability in the left potential well, and, conversely, the negative correlation will increase the amount of probability in the right potential well.

However, our results may provide some theoretical intuitions for real gene expression. For example, Hasty et al. [3] investigated the autoregulation of 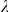 repressor expression in the lysis-lysogeny pathway in the 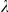 virus, and they showed why the additive and multiplicative noises can be used to regulate expression (or to control the protein production). Our results further show how the probability transition between two stable states (i.e. two levels of protein synthesis) is affected, and why the cross-correlation between the additive and multiplicative external noises can be also used to regulate expression. Finally, we would like to say that the further analysis incorporating more realistic dynamics should be carried out in future studies.

## Supporting Information

Text S1 Steady-state statistics, Probability transition rate, First-passage time, Method for stochastic simulation.
(PDF)
