# Supplementary material for: Bistability, Probability Transition Rate and First-Passage Time in an Autoactivating Positive-Feedback Loop
Source: PLoS One. 2011 Mar 21;6(3):e17104. doi: 10.1371/journal.pone.0017104 (PMC3061858; doi:10.1371/journal.pone.0017104)
Supplement: Text S1 — Steady-state statistics, Probability transition rate, First-passage time, Method for stochastic simulation. (PDF) [file pone.0017104.s001.pdf]

Text Supplementary 1— Bistability, probability transition rate and first-passage time in an autoactivating positive-feedback loop

Xiu-Deng Zheng[1,3], Xiao-Qian Yang[2] and Yi Tao[1,*]
1 Key Laboratory of Animal Ecology and Conservational Biology
Centre for Computational and Evolutionary Biology
Institute of Zoology, Chinese Academy of Sciences
Beijing, P.R. China
2 School of Mathematical Sciences, Beijing Normal University
Beijing, P.R. China
3 Graduate University of the Chinese Academy of Sciences
Beijing, P.R. China
$*$ Author for correspondence, e-mail: yitao@ioz.ac.cn

Steady-state statistics

For convenience, let $\tilde{y} = y - y_{s_i}$. Then, the distribution of $\tilde{y}$ is given by

$$\psi(\tilde{y}, t) = \left(d\tilde{y}/dy\right)^{-1} \phi(y, t) \; . \tag{S1}$$

Obviously, $\psi(\tilde{y}, t) = \phi(y, t)$ since $d\tilde{y}/dy = 1$. Thus, from Eq. 8, we have

$$\frac{\partial \psi(\tilde{y}, t)}{\partial t} = -\frac{\partial}{\partial \tilde{y}} \, G(\tilde{y}) \, \psi(\tilde{y}, t) + D_A \frac{\partial^2}{\partial \tilde{y}^2} \, \psi(\tilde{y}, t) \; , \tag{S2}$$

where $G(\tilde{y}) = g(\tilde{y} + y_{si})$. When $\tilde{y}$ is near 0, we have

$$\begin{aligned} \frac{d\langle \tilde{y} \rangle}{dt} &= \int_{-\infty}^{\infty} \tilde{y} \, \frac{\partial \psi(\tilde{y}, t)}{\partial t} \, d\tilde{y} \\ &\approx \int_{-\infty}^{\infty} \tilde{y} \left[-\frac{\partial}{\partial \tilde{y}} \, G'(0) \tilde{y} \psi(\tilde{y}, t) + D_A \frac{\partial^2}{\partial \tilde{y}^2} \, \psi(\tilde{y}, t) \right] d\tilde{y} \\ &= G'(0) \langle \tilde{y} \rangle \end{aligned} \tag{S3}$$

with $G'(0) = g'(y_{s_i}) < 0$, and

$$\begin{aligned} \frac{d\langle \tilde{y}^2 \rangle}{dt} &= \int_{-\infty}^{\infty} \tilde{y}^2 \, \frac{\partial \psi(\tilde{y}, t)}{\partial t} \, d\tilde{y} \\ &\approx \int_{-\infty}^{\infty} \tilde{y}^2 \left[-\frac{\partial}{\partial \tilde{y}} \, G'(0) \tilde{y} \psi(\tilde{y}, t) + D_A \frac{\partial^2}{\partial \tilde{y}^2} \, \psi(\tilde{y}, t) \right] d\tilde{y} \\ &= 2G'(0) \langle \tilde{y}^2 \rangle + 2D_A \; . \end{aligned} \tag{S4}$$

Hence, for large time t, we have $\langle \tilde{y} \rangle = 0$ and $\langle \tilde{y}^2 \rangle = -D_A/g'(y_{s_i})$, i.e., $\langle y \rangle = y_{s_i}$ and $\langle y^2 \rangle = -D_A/g'(y_{s_i}) + y_{s_i}^2$.

Probability transition rate

Similar to the analysis in the steady-state statistics, let $z = y - y_u$ with $z_{s_i} = y_{s_i} - y_u$ for $i = 1, 2$ and $z_u = 0$. The distribution of z is given by

$$\varphi(z,t) = \left(dz/dy\right)^{-1} \phi(y,t) \tag{S5}$$

with $dz/dy = 1$. Similar also to Eq. S2, we have

$$\frac{\partial \varphi(z,t)}{\partial t} = -\frac{\partial}{\partial z} S(z)\varphi(z,t) + D_A \frac{\partial^2}{\partial z^2} \varphi(z,t) \ , \tag{S6}$$

where $S(z) = g(z + y_u)$. According to Eq. 9, the potential function can be given by

$$U_{FP}(z) = \frac{1}{2} \ln D_A - \int^z \frac{S(s)}{D_A} \, dz \tag{S7}$$

with $U'_{FP}(z) = -S(z)/D_A$. From van Kampen [1] and Hu [2], the Gaussian approximation of $\varphi(z,t)$ is

$$\varphi(z,t) \approx \frac{1}{\sqrt{2\pi D_A \sigma(t)}} \exp\left[-\frac{(z - z(t))^2}{2 D_A \sigma(t)} \right] , \tag{S8}$$

where $z(t)$ and $\sigma(t)$ are the solution of equation

$$\begin{aligned} \frac{dz(t)}{dt} &= S(z(t)) \ , \\ \frac{d\sigma(t)}{dt} &= 2S''(z(t))\sigma(t) + 2 \ . \end{aligned} \tag{S9}$$

Before the system approaches the stationary distribution, the probability distribution of the quasi-stable state can be expressed as

$$\varphi(z,t) = \begin{cases} \varphi_-(z,t) = N_-(t) e^{-U_{FP}(z)} & , \ \text{if } z \le 0 \\ \varphi_+(z,t) = N_+(t) e^{-U_{FP}(z)} & , \ \text{if } z > 0 \end{cases} \tag{S10}$$

where both $N_+(t)$ and $N_-(t)$ are functions of time t such that

$$\int_{-\infty}^{0} \varphi_-(z,t) dz + \int_{0}^{+\infty} \varphi_+(z,t) dz = 1 \ .$$

Notice that the probability distribution has two sharp probability peaks around z_{s_1} and z_{s_2}, and the Taylor expansion of $U_{FP}(z)$ about z_{s_i} is

$$U_{FP}(z) = U_{FP}(z_{s_i}) + \frac{1}{2} U''_{FP}(z_{s_i})(z - z_{s_i})^2 \ . \tag{S11}$$

Hence, the total probability in the left well (region for all $z \leq 0$) can be given by

$$P_-(t) \;=\; \int_{-\infty}^{0} \varphi_-(z,t)\; dz = N_-(t)\sqrt{2\pi/U''_{FP}(z_{s_1})}\; e^{-U_{FP}(z_{s_1})}\;, \tag{S12}$$

and, similarly, the total probability in the right well (region for all $z > 0$), is

$$P_+(t) \;=\; \int_{0}^{+\infty} \varphi_+(z,t)\; dz = N_+(t)\sqrt{2\pi/U''_{FP}(z_{s_2})}\; e^{-U_{FP}(z_{s_2})}\;, \tag{S13}$$

On the other hand, the change rate of $P_-(t)$ can be expressed as

$$\begin{aligned} \frac{dP_-(t)}{dt} &= \int_{-\infty}^{0} \frac{\partial \varphi_-(z,t)}{\partial t}\; dz \\ &= \int_{-\infty}^{0} \left[-\frac{\partial}{\partial z}\; S(z)\varphi(z,t) + D_A \frac{\partial^2}{\partial z^2}\varphi(z,t) \right\}\, dx \\ &= D_A \frac{\partial}{\partial z}\; \varphi(z,t)\Big|_{z=0}\;. \end{aligned} \tag{S14}$$

This shows clearly that the change rate of $P_-(t)$ depends only on the properties of $\varphi(z,t)$ at $z = 0$. Similarly, the change rate of $P_+(t)$ is

$$\frac{dP_+(t)}{dt} \;=\; -D_A \frac{\partial}{\partial z}\; \varphi(z,t)\Big|_{z=0}\;. \tag{S15}$$

In order to determine the probability current from the left well to the right well, we can assume that at time t the initial distribution is

$$\varphi_{in}(z,t) \;=\; \begin{cases} \varphi_-(z,t)\;, & \text{if} \quad z \leq 0 \\ 0\;, & \text{if} \quad z > 0 \end{cases} \tag{S16}$$

From the Ω-expansion of the Green function [2], $\varphi(z, t+ \vartriangle t)$ at time can be expressed as

$$\varphi(z,t+\vartriangle t) \;=\; \int_{-\infty}^{0} \varphi_\Omega(z,t+\vartriangle t)\; \varphi_{in}(s,t)\; ds\;, \tag{S17}$$

where (i) $\varphi_\Omega(z, t+ \vartriangle t)$ is the Gaussian approximation of $\varphi(z, t+ \vartriangle t)$, and when z is near 0, $\varphi_\Omega(z, t+ \vartriangle t)$ can be expressed as

$$\varphi_\Omega(z,t+\vartriangle t) \;=\; \sqrt{\frac{\alpha}{2\pi D_A(e^{2\alpha\Delta t}-1)}}\,\exp\left[\frac{-\alpha(z - z(t)e^{\alpha\Delta t})^2}{2D(e^{2\alpha\Delta t}-1)}\right], \tag{S18}$$

where $\alpha = |S'(0)|$; and (ii) $\varphi_{in}(s,t)$ is given by

$$\varphi_{in}(s,t) \;=\; k_- P_-(t) e^{\alpha s^2/2D_A}\;, \tag{S19}$$

for $s \leq 0$, where

$$k_- \;=\; \sqrt{\frac{U''_{FP}(z_{s_1})}{2\pi}}\; e^{U_{FP}(z_{s_1}) - U_{FP}(0)}\;. \tag{S20}$$

Thus, we have

$$\begin{aligned}
\varphi(z, t+\bigtriangleup t) &= k_- P_-(t) \sqrt{\frac{\alpha}{2\pi D_A (e^{2\alpha\Delta t}-1)}} \int_{-\infty}^{0} \exp\left[-\frac{\alpha(z-se^{\alpha\Delta t})^2}{2D_A(e^{2\alpha\Delta t}-1)}\right] \exp\left[\frac{\alpha s^2}{2D_A}\right] ds \\
&= k_- P_-(t) \sqrt{\frac{\alpha}{2\pi D_A (e^{2\alpha\Delta t}-1)}} \int_{0}^{+\infty} \exp\left[-\frac{\alpha(z+se^{\alpha\Delta t})^2}{2D_A(e^{2\alpha\Delta t}-1)} + \frac{\alpha s^2}{2D_A}\right] ds \\
&= k_- P_-(t) \sqrt{\frac{\alpha}{2\pi D_A (e^{2\alpha\Delta t}-1)}} \int_{0}^{+\infty} \exp\left[-\frac{\alpha[s^2+2sze^{\alpha\Delta t}+z^2]}{2D_A(e^{2\alpha\Delta t}-1)}\right] ds \\
&= k_- P_-(t) \sqrt{\frac{\alpha}{2\pi D_A (e^{2\alpha\Delta t}-1)}} \int_{0}^{+\infty} \exp\left[-\frac{\alpha[s+ze^{\alpha\Delta t}]^2}{2D_A(e^{2\alpha\Delta t}-1)} + \frac{\alpha z^2}{2D_A}\right] ds \\
&\approx k_- P_-(t) \frac{1}{\sqrt{\pi}} \int_{\sqrt{\frac{\alpha}{2D_A(e^{2\alpha\Delta t}-1)}}\, ze^{\alpha\Delta t}}^{+\infty} e^{-s^2}\, ds \\
&= k_- P_-(t) \frac{1}{\sqrt{\pi}} \left[\frac{\sqrt{\pi}}{2} - \int_{0}^{\sqrt{\frac{\alpha}{2D_A(e^{2\alpha\Delta t}-1)}}\, ze^{\alpha\Delta t}} e^{-s^2}\, ds\right] \\
&\approx k_- P_-(t) \left[\frac{1}{2} - \sqrt{\frac{\alpha}{2\pi D_A(e^{2\alpha\Delta t}-1)}}\, ze^{\alpha\Delta t}\right] \\
&\approx k_- P_-(t) \left[\frac{1}{2} - \sqrt{\frac{\alpha}{2\pi D_A}}\, z\right] ,
\end{aligned} \tag{S21}$$

where we use the conditions: (i) $|z|$ is small, and the terms $|z|^n$ are ignored if $n \geq 2$; (ii) $\int_0^x e^{-t^2} dt = x - \frac{1}{3}x^3 + O(x^5)$ when $|x| \ll 1$; (iii) $D_A^{-1/2} \gg e^{\alpha\Delta t} \gg 1$. Hence, the change rate of $P_-(t)$ can be expressed as

$$\frac{dP_-(t)}{dt} = -R_- P_-(t) ,$$

where the parameter R_- is the probability transition rate from the left well to the right well, which is given by

$$\begin{aligned}
R_- &= D_A k_- \sqrt{\frac{\alpha}{2\pi D_A}} \\
&= \frac{D_A}{2\pi} \sqrt{U''_{FP}(z_{s_1})|U''_{FP}(0)|}\; e^{U_{FP}(z_{s_1})-U_{FP}(0)} .
\end{aligned} \tag{S22}$$

Similarly, if the total probability initially distributes in the right well, the change rate of $P_+(t)$ can be expressed as

$$\frac{dP_+(t)}{dt} = -R_+ P_+(t) , \tag{S23}$$

where the parameter R_+ is the probability transition rate from the right well to the left well, which is given by

$$R_+ = \frac{D_A}{2\pi} \sqrt{|U''_{FP}(0)|U''_{FP}(z_{s_2})}\; e^{U_{FP}(z_{s_2})-U_{FP}(0)} . \tag{S24}$$

First-passage time

Similar to the analysis in the probability transition rate, let $z = y - y_u$ with $z_{s_i} = y_{s_i} - y_u$ for $i = 1, 2$ and $z_u = 0$. We first consider the statistical properties of the first-passage time τ_-. The analysis in the following is mainly from Risken [3]. Let $\varphi(z, t|z_0, 0)$ denote the probability density that the system state reaches z at time t with initial z_0 at $t = 0$ (where $z,\ z_0 < 0$). In order to understand how the system state escapes from the left well completely, we introduce a given boundary A with $A > 0$ and assume A to be an absorbing wall (i.e., when we consider the first-passage time τ_-, we temporarily ignore the state transition from the right well to left well, or the system states are no longer counted if they have passed A). For $z < A$, we have

$$\begin{aligned}
&\frac{\partial \varphi}{\partial t} = L_{FP}\varphi \ ; \\
&\varphi(z, 0|z_{s_1}, 0) = \delta(z - z_{s_1}) \quad \text{for } z < A \ , \\
&\varphi(A, t|z_{s_1}, 0) = 0 \ .
\end{aligned} \tag{S25}$$

where L_{FP} is the Fokker-Planck operator, which is

$$\begin{aligned}
L_{FP} &= D_A \frac{\partial}{\partial z}\, U'_{FP}(z) + D_A \frac{\partial^2}{\partial z^2} \\
&= D_A \frac{\partial}{\partial z}\, e^{-U_{FP}(z)}\, \frac{\partial}{\partial z}\, e^{U_{FP}(z)} \ .
\end{aligned} \tag{S26}$$

Here we use $W(z_{s_1}, t)$ to denote the total probability in the left well at time t, or the probability of realizations which started at z_{s_1} and not yet reached the boundary A at time t, i.e., we have

$$W(z_{s_1}, t) \ = \ \int_{-\infty}^{A} \varphi(z, t|z_{s_1}, 0)dx \ .$$

The probability of those realizations which reach the boundary A in the time interval $(t, t + dt)$, denoted by $-dW$, is

$$-dW(z_{s_1}, t) \ = \ -\int_{-\infty}^{A} \dot{\varphi}(z, t|z_{s_1}, 0)dx\ dt \ .$$

This implies that the distribution of τ_-, denoted by $w(z_{s_1}, \tau_-)$, can be given by

$$w(z_{s_1}, \tau_-) = -\frac{dW(z_{s_1}, \tau_-)}{d\tau_-} = -\int_{-\infty}^{A} \dot{\varphi}(z, \tau_-|z_{s_1}, 0)dz \ .$$

Thus, the n moments of τ_- (for $n = 0, 1, 2, \cdots$) are

$$\langle \tau_-^n \rangle = \int_0^{\infty} \tau_-^n w(z_{s_1}, \tau_-)d\tau_- = \int_{-\infty}^{A} \vartheta_n(z, z_{s_1})dx \ .$$

where $\vartheta_n(x, x_{s_1})$ is defined as

$$\vartheta_n(z, z_{s_1}) \ = \ -\int_0^{\infty} \tau_-^n \dot{\varphi}(z, \tau_-|z_{s_1}, 0)d\tau_- \ .$$

Then, we have

$$\vartheta_0(z, z_{s_1}) \;=\; -\int_0^\infty \dot{\varphi}(z, \tau_-|z_{s_1}, 0)d\tau_- = \delta(z - z_{s_1}) \ .$$

and

$$\vartheta_n(z, z_{s_1}) = n\int_0^\infty \tau_-^{n-1}\varphi(z, \tau_-|z_{s_1}, 0)d\tau_- \ , \quad \text{for } n \geq 1 \ .$$

By applying the operate L_{FP} to the above equation, we obtain

$$\begin{aligned} L_{FP}\vartheta_n(z, z_{s_1}) &= n\int_0^\infty \tau_-^{n-1} L_{FP}\varphi(z, \tau_-|z_{s_1}, 0)d\tau_- \\ &= n\int_0^\infty \tau_-^{n-1}\dot{\varphi}(z, \tau_-|z_{s_1}, 0)d\tau_- \\ &= -n\vartheta_{n-1}(z, z_{s_1}) \ , \end{aligned}$$

i.e.,

$$\begin{aligned} L_{FP}\vartheta_1(z, z_{s_1}) &= -\delta(z - z_{s_1}) \ , \\ L_{FP}\vartheta_2(z, z_{s_1}) &= -2\vartheta_1(z, z_{s_1}) \ , \\ L_{FP}\vartheta_3(z, z_{s_1}) &= -3\vartheta_2(z, z_{s_1}) \ , \\ \cdots &= \cdots \ . \end{aligned} \tag{S27}$$

From above equations, we can obtain ϑ_n, which are

$$\vartheta_n(z, z_{s_1}) = n![-L_{FP}(z)]^{-n}\delta(z - z_{s_1}) \tag{S28}$$

with boundary conditions $\vartheta_n(A, z_{s_1}) = 0$ for $n \geq 1$, where $[-L_{FP}(z)]^{-n}$ denotes the inverse operator of $[-L_{FP}(z)]^n$.

Notice that for $n = 1$, we have that

$$\begin{aligned} \vartheta_1(z, z_{s_1}) &= D_A^{-1} e^{-U_{FP}(z)} \int_z^A e^{U_{FP}(y)} \left[\int_{-\infty}^y \delta(x - z_{s_1})dx\right] dy \\ &= D_A^{-1} e^{-U_{FP}(z)} \int_z^A e^{U_{FP}(y)} dy \ . \end{aligned}$$

Thus,

$$\langle \tau_- \rangle \;=\; D_A^{-1} \int_{-\infty}^A e^{-U_{FP}(z)} \left[\int_z^A e^{U_{FP}(y)} dy\right] dz \ . \tag{S29}$$

For the weak noise $D_A \ll 1$, the region about z_{s_1} contributes mostly to the first integral in Eq. S29 where $U_{FP}(z)$ can be replaced by its Taylor expansion about $z = z_{s_1}$

$$U_{FP}(z) \;=\; U_{FP}(z_{s_1}) + \frac{1}{2}U''_{FP}(z_{s_1})(z - z_{s_1})^2 \ ,$$

and the region about $z_u = 0$ contributes mostly to the second integral where $U_{FP}(z)$ can be replaced by

$$U_{FP}(z) = U_{FP}(0) - \frac{1}{2}|U''_{FP}(0)|z^2.$$

Therefore, Eq. S29 can be rewritten as

$$\langle \tau_- \rangle \approx \frac{2\pi}{D_A\sqrt{U''_{FP}(z_{s_1})|U''_{FP}(0)|}} e^{U_{FP}(0)-U_{FP}(z_{s_1})} . \tag{S30}$$

Similarly, we have also

$$\begin{aligned}\vartheta_2(z, z_{s_1}) &= 2D_A^{-1}e^{-U_{FP}(z)}\int_z^A e^{U_{FP}(y)}\left[\int_{-\infty}^y \vartheta_1(x, z_{s_1})dx\right]dy \\ &= 2D_A^{-2}e^{-U_{FP}(z)}\int_z^A e^{U_{FP}(y)}\left\{\int_{-\infty}^y e^{-U_{FP}(x)}\left[\int_x^A e^{U_{FP}(w)}dw\right]dx\right\}dy ,\end{aligned}$$

i.e.,

$$\begin{aligned}\langle \tau_-^2 \rangle &= 2D_A^{-2}\int_{-\infty}^A e^{-U_{FP}(z)}\int_z^A e^{U_{FP}(y)}\left\{\int_{-\infty}^y e^{-U_{FP}(x)}\left[\int_x^A e^{U_{FP}(w)}dw\right]dx\right\}dydz \\ &\approx \frac{8\pi^2}{D_A^2 U''_{FP}(z_{s_1})|U''_{FP}(0)|}e^{2[U_{FP}(0)-U_{FP}(z_{s_1})]} \\ &= 2\langle \tau_- \rangle^2 .\end{aligned}$$

Thus, the variance of τ_- is given by

$$V_{\tau_-} = \langle \tau_-^2 \rangle - \langle \tau_- \rangle^2 \approx \langle \tau_- \rangle^2 .$$

From Eq. S22, we have

$$\begin{aligned}\langle \tau_- \rangle &\approx 1/R_- , \\ V_{\tau_-} &\approx 1/R_-^2 .\end{aligned} \tag{S31}$$

Similarly, for the statistical properties of τ_+, we have

$$\begin{aligned}\langle \tau_+ \rangle &\approx 1/R_+ , \\ V_{\tau_+} &\approx 1/R_+^2 .\end{aligned} \tag{S32}$$

For the calculation of the first-passage time in Eq. 17, we need a suitable transformation, in which the y-dependent diffusion coefficient can be transformed to an arbitrary constant $D > 0$. We use the transformation

$$s = s(y) = \int^y \sqrt{D/D^{(2)}(\xi)}d\xi . \tag{S33}$$

Then the transformed drift coefficient and diffusion coefficient are taken as

$$\begin{aligned}
D'^{(1)} &= \frac{ds}{dy}D^{(1)}(y) + \frac{d^2s}{dy^2}D^{(2)}(y) \ , \\
&= \sqrt{\frac{D}{D^{(2)}(y)}}\big[D^{(1)}(y) - \frac{1}{2}\frac{dD^{(2)}(y)}{dy}\big] \ , \\
D'^{(2)} &= D = \big(\frac{ds}{dy}\big)^2 D^{(2)}(y) \ , \qquad \text{(S34)}
\end{aligned}$$

where D is an arbitrary constant. The transformed Fokker-Planck equation (FPE) reads

$$\frac{\partial\varphi(s,t)}{\partial t} = -\frac{\partial}{\partial s}D'^{(1)}(s)\varphi(s,t) + D\frac{\partial^2}{\partial s^2}\varphi(s,t) \ , \qquad \text{(S35)}$$

where $\varphi(s,t)$ is given by

$$\varphi(s,t) = \left(\frac{ds}{dy}\right)^{-1}\phi(y,t) = \sqrt{D^{(2)}(y)/D}\phi(y,t) \qquad \text{(S36)}$$

(see Ref. [3]). Therefore, from Eqs. S30 and S33, it is easy to obtain Eq. 17.

Method for stochastic simulation

The numerical integration of a Langevin equation (LE) is intrinsically different from that of an ordinary differential equation. Several different algorithms have been proposed recently to get the numerical solution of LE [3-5]. The main idea of our algorithms is from Denisov et al.[4,5].

To obtain a discretized version of Eq. 4, we choose a small time interval Δ. The value δy is given by

$$\delta y = g(y(t'))\Delta + D_A^{1/2}\delta W_1 + D_M^{1/2}h(y(t'))\delta W_2 \qquad \text{(S37)}$$

Here $\delta y = y(t+\Delta) - y(t)$, $t' \in [t, t+\Delta]$, and $\delta W_i = W_i(t+\Delta) - W_i(t)$ are the increments of Wiener processes $W_i(t)$ satisfying the conditions

$$< \delta W_i >= 0, \quad < \delta W_i \delta W_i >= 2\Delta, \quad < \delta W_1 \delta W_2 >= 2\mu\Delta$$

for $i = 1, 2$ respectively.

Since $\delta W_i \propto \Delta^{1/2}$ and $y(t') \approx y(t) + \delta y/2$, we obtain the formula

$$\begin{aligned}
\delta y \approx\ & g(y(t))\Delta + D_A^{1/2}\delta W_1 + D_M^{1/2}h(y(t))\delta W_2 \\
& + D_M h'(y(t))h(y(t))(\delta W_2)^2/2 + (D_A D_M)^{1/2}h(y(t))\delta W_1 \delta W_2/2 \qquad \text{(S38)}
\end{aligned}$$

which is accurate to the first order of Δ. Therefore, the numerical solution of the LE can be calculated according to

$$\begin{aligned}
y(t+\Delta) \approx\ & y(t) + g(y(t))\Delta + D_A^{1/2}\Delta^{1/2}\omega_1 + D_M^{1/2}h(y(t))\Delta^{1/2}\omega_2 \\
& + D_M h'(y(t))h(y(t))\Delta\omega_2^2/2 + (D_A D_M)^{1/2}h(y(t))\Delta\omega_1\omega_2/2 \qquad \text{(S39)}
\end{aligned}$$

Here ω_1 and ω_2 are Gaussian-distributed random variables with

$$< \omega_i >= 0, \quad < \omega_i^2 >= 2, \quad < \omega_1 \omega_2 >= 2\mu$$

for $i = 1, 2$ respectively.

References

[1] van Kampen NG (1992) *Stochastic Process Theory in Physics and Chemistry.* North-Holland, Amsterdam.

[2] Hu G (1992) *Stochastic Forces and Nonlinear System.* Shanghai Press for Science, Technology and Education (Chinese version).

[3] Risken H (1992) *The Fokker-Planck Equation: Methods of Solution and Applications.* Springer, Berlin.

[4] Denisov SI, Virenko AN, Horsthemke W (2003) Nonequilibrium transitions induced by the cross-correlation of white noise. Phys Rev E 68: 046132.

[5] Sancho JM, San Miguel M, Katz SL, Gunton JD (1982) Analytical and numberical studies of multiplicative noise. Phys Rev A 26: 1589-1609.
